# Adult Vascular Wall Resident Multipotent Vascular Stem Cells, Matrix Metalloproteinases, and Arterial Aneurysms

**DOI:** 10.1155/2015/434962

**Published:** 2015-03-18

**Authors:** Bruno Amato, Rita Compagna, Maurizio Amato, Raffaele Grande, Lucia Butrico, Alessio Rossi, Agostino Naso, Michele Ruggiero, Stefano de Franciscis, Raffaele Serra

**Affiliations:** ^1^Interuniversity Center of Phlebolymphology (CIFL), International Research and Educational Program in Clinical and Experimental Biotechnology, Magna Graecia University of Catanzaro, Viale Europa, 88100 Catanzaro, Italy; ^2^Department of Clinical Medicine and Surgery, University of Naples “Federico II”, 80100 Naples, Italy; ^3^Department of Medical and Surgical Sciences, University of Catanzaro, 88100 Catanzaro, Italy; ^4^Department of Medicine and Health Sciences, University of Molise, 88100 Campobasso, Italy

## Abstract

Evidences have shown the presence of multipotent stem cells (SCs) at sites of arterial aneurysms: they can differentiate into smooth muscle cells (SMCs) and are activated after residing in a quiescent state in the vascular wall. Recent studies have implicated the role of matrix metalloproteinases in the pathogenesis of arterial aneurysms: in fact the increased synthesis of MMPs by arterial SMCs is thought to be a pivotal mechanism in aneurysm formation. The factors and signaling pathways involved in regulating wall resident SC recruitment, survival, proliferation, growth factor production, and differentiation may be also related to selective expression of different MMPs. This review explores the relationship between adult vascular wall resident multipotent vascular SCs, MMPs, and arterial aneurysms.

## 1. Introduction

The vascular wall is composed of a limited number of different mesodermic cells, endothelial cells (ECs), smooth muscle cells (SMCs), and adventitial stromal fibroblasts. Recent studies have indicated that the human arterial wall also contains resident progenitor cell with angiogenetic properties, known as vascular wall resident progenitor cells (VW-PCs) [1, 2]. These cells arise during embryonic and fetal age but still remain niched and functional in the adult to guarantee the renewal and repair of vascular tissue and trigger the processes of “postnatal angiogenesis” [3].

Angiogenesis, characterized by the growth of new blood vessels or capillaries from preexisting vessels, plays a pivotal role in the postnatal tissue remodeling both in physiological and in pathological conditions [4]. In this way, studies have shown that matrix metalloproteinases (MMPs) are involved in the degradation of the extracellular matrix (ECM) substrates regulating structural proteins and consequent tissue remodeling and may be considered potential early biomarkers of evolution of vascular and nonvascular disease. But MMPs play a regulatory role and participate in key stages of postnatal angiogenesis as follows: the endothelial proliferation and migration, tub formation with an encased lumen sealed by tight cell–cell junctions, synthesis of ECM proteins, and the recruitment of mural cells stabilizing new connections [5].

Evidences have shown the presence of multipotent stem cells (SCs) at sites of arterial aneurysms; they can differentiate into SMCs and are activated after residing in a quiescent state in the vascular wall [6–8]. The factors and signaling pathways involved in regulating wall resident SC recruitment, survival, proliferation, growth factor production, and differentiation may be also related to selective expression of different MMPs [9–11].

The purpose of this review is to examine the role of vascular wall resident stem cells and biomolecular mechanisms that regulate the activity of MMPs in natural history of arterial aneurysms.

## 2. Materials and Methods

PubMed and ScienceDirect databases were searched for articles using the terms adult vascular wall resident stem cells, angiogenesis, MMPs, arterial aneurysms, and chronic inflammation.

Only publications in English were included. Titles and abstracts were screened by 3 authors (Michele Ruggiero, Agostino Naso, and Stefano de Franciscis) to identify potentially relevant studies. All potentially eligible studies were subsequently evaluated in detail by 1 reviewer and 3 authors (Michele Ruggiero, Agostino Naso, and Stefano de Franciscis) through consideration of the full text. Reference lists of retrieved articles were also searched for relevant publications.

Clinical trial, meta-analysis, multicenter study, review, and systematic reviews published in the last 5 years were included. Studies were excluded if they were not in English language, if performed in vitro, if the cohort was defined by the presence of arterial aneurysms and an additional confounding disease process (e.g., chronic renal failure or cerebrovascular diseases), or if arterial aneurysms specific results could not be distinguished from those of a larger population consisting of individuals without disease. Studies were excluded when the primary focus was carotid artery disease, inflammatory diseases, cancer, nonvascular diseases, and treatment with chemotherapy.

## 3. Results

### 3.1. Study Selection

**Figure 1:**
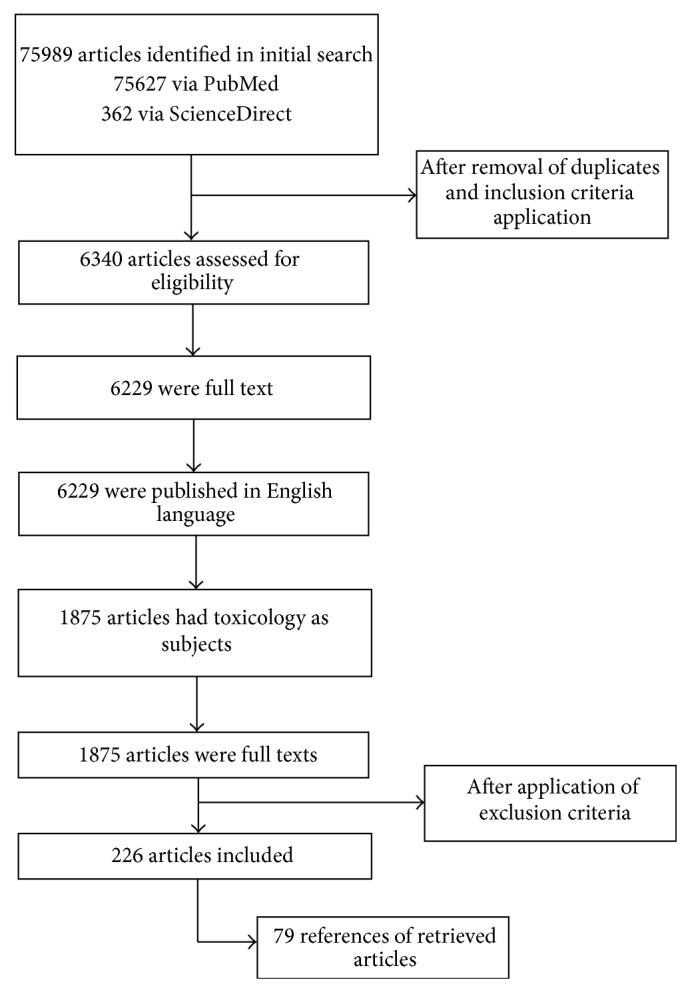
Flow of papers identified from search strategy.

Initial database searches yielded 75627 studies from PubMed and 362 from Science Direct in the last 5 years. We evaluated 1875 eligible full text articles (Figure 1).

The biology and physiology of vascular wall resident stem cells and their role in postnatal angiogenesis, the current evidences on MMPs activity and their correlation with various stages of angiogenesis, the relationship with MMPs and arterial aneurismal disease, and the association between MMPs, arterial aneurysms, and physiology of vascular wall resident stem cells are given below.

#### 3.1.1. Adult Vascular Wall Resident Stem Cells and Angiogenesis

Many evidences have shown that fetal and adult arterial and venous vessel walls may be niches for various stem and progenitor cells, such as endothelial progenitor cells (EPCs), smooth muscle cell (SMC) progenitors, hematopoietic stem cells (HSCs), mesenchymal stem cells (MSCs), and the so-called mesangial cells, coexpressing both endothelial and myogenic markers [12–15]. Zengin et al. identified VW-PC in human arteries and veins, characterised by expression of CD34+, vascular endothelial growth factor receptor-2 (VEGFR2), and tyrosine kinase with immunoglobulin-like and EGF-like domains 2 (TIE2) and were found in the region between the media and adventitia. These cells have been found in different layers (intima, media, and adventitia) and they can differentiate into ECs and contribute to new vessel formation in both physiological and pathologic condition [9, 15–18]. The wall of adult human blood vessels harbours contains not only EPCs but also CD44(+) CD34(−) CD45(−) multipotent MSC-like stem cells, which are capable of differentiating into pericytes/SMC and covering endothelial cell layers of newly formed blood vessels in vitro and in vivo [19]. This zone was identified in human adult vessels as a niche for CD34+ CD31− EPCs and for progenitors of macrophages earlier. Later, it was shown that CD34(+) Sca1(+) cells cluster in a domain of Sonic hedgehog signaling which was restricted to the inner part of mouse arterial adventitia similar to the vasculogenic zone [20]. Vasculogenic zone in the wall of vessels acts as a source of progenitor cells and is in relation to those of EPCs circulating in peripheral blood or derived from the bone marrow [21] but it also serves as a reservoir for inflammatory cells important for local immune response. VW-PCs reside in this zone from the developmental embryonic to adult phase and have the capacity to differentiate into SMC and pericytes and are able to form capillary sprouts and migrate towards angiogenic lineage [18].

Vasculogenesis is defined as de novo vessel formation induced by differentiation of angioblasts and it is the major mechanism of formation of blood island vessels, dorsal aorta, endocardium, and vitelline vessels in the embryo. Angiogenesis is defined as outgrowth of new vessels from preexisting blood vessels and vascular growth and remodeling are key events in the adaptation of arteries to physiological and pathological environmental stimuli [22]. Several steps of this process are endothelial cell migration, proliferation, and tube formation [23–25].

VW-PCs normally involved in physiological vascular homeostasis might also act as reservoir of undifferentiated cells ready to supply the cellular demands and acquiring local phenotypic characteristics [26]. The active cellular component in these processes is granted by endothelial lineage cells, but neovascularization does not only depend on endothelial cell migration and proliferation with subsequent formation of endothelial tubes; it also requires pericyte coverage of vascular sprouts for vessel stabilization and survival; these cells were capable of differentiating into vascular SMCs and pericytes under in vitro and in vivo conditions [27]. MSCs may represent an important source of pericytes and SMCs during angiogenesis under physiological and pathological conditions. Evidences show that these cells migrate to the vascular injury sites in postnatal life to replace dead or dysfunctional cells [28–31].

#### 3.1.2. Vascular Wall Resident Cells and Aneurysms

Aneurysmal disease is one of the most common clinical diseases in Western countries [32] and is related to the presence of multiple risk factors such as alterations of glucose and lipid metabolism, hypertension, trauma, anastomotic disruption, infections, and connective or inflammatory diseases. As described previously [32], arterial aneurysms can be divided into central aneurysms, such as abdominal aortic aneurysms, and peripheral aneurysms, such as aneurysms of the popliteal, femoral, and carotid arteries [32].

Arterial aneurysms are caused by two combined mechanisms that lead to progressive medial degeneration and vessel dilation: increased degradation by MMPs [33] and decreased synthesis of elastin caused by apoptosis of vascular SMCs [34]. Moreover, chronic inflammation and consequent oxidative stress promote progressive vascular wall impairment [35]. As described above, recent studies have shown that the wall of adult blood vessels itself can be considered as reservoir for resident stem cells [18, 36, 37]. These VW-PCs largely reside in the “vasculogenic” area giving birth to generation of pericytes/SMCs which are involved in the formation of new vessels and can be activated by endothelial injuries or other vascular insults undergoing changes that include proliferation, differentiation, and migration [38, 39]. VW-PCs could aggregate at sites of injury and differentiate into ECs or move across vascular wall towards the intima and differentiate into SMCs [40]. Moreover, differentiation and behaviour of VW-PCs are regulated by adventitia through releasing factors involved in the regulation of wall functions [40]. In many conditions, such as presence of atherosclerotic plaques or injury, resident stem cells are activated and stimulated to acquire specific structural and functional behaviour [41, 42], so the vasculogenic area is thought to be also a niche of undifferentiated cells acquiring specific phenotypic characteristics and during the development of pathologic conditions affecting the vessel walls [41]. In order to fulfill their duties, these cells have to be mobilized and released from their niches. Some studies suggest that specific inflammation of adventitia leads to the production of cytokines or enzymes such as tumor necrosis factor alpha (TNF-*α*), transforming growth factor beta (TGF-*β*), granulocyte colony stimulating factor (G-CSF), granulocyte macrophage colony stimulating factor (GM-CSF), monocyte chemoattractant protein-1 (MCP-1), and stromal cell derived factor 1-alpha (SDF1-*α*), all factors able to promote SCs mobilization towards sites of injury via vasa vasorum [41–46]. The relation between arterial aneurysms and VW-PC is hypothesized. Ryer et al. described a possible proinflammatory role of stem cells in abdominal aortic aneurysms and it was observed in infrarenal aortic wall specimens collected from patients with abdominal arterial aneurysms (AAA) undergoing surgical repair; a significantly great number of c-kit+ and CD34+ cells also express macrophage marker CD68 but not the SMCs marker SM22 or the fibroblast marker FSP1. Moreover CD68+ cells colocalized with the cellular marker of proliferation Ki67 [36]. These findings suggest an inflammatory/immune role of resident stem cells in AAA pathogenesis and were also confirmed by other authors [47, 48].

Studies showed that altered hemodynamical forces probably affect resident stem cells differentiation. In particular, shear stress can stimulate these resident stem cells to differentiate into endothelial lineage whereas cyclic strain leads to smooth muscle differentiation. So disturbed blood flow and distorted biomechanical stress can lead to abnormal differentiation of vascular stem cells whose altered behaviour may lead to the development of vascular wall diseases, such as arterial aneurysms [39].

### 3.2. Biology of MMPs

MMPs, a group of zinc dependent proteinases consisting of 28 family members, play important roles in ECM degradation as well as in the cleavage of other proteins such as growth factor and cytokines [49] and it is critical for all aspects of vascular biology [50]. Serra et al. have shown that MMPs are implicated in main vascular diseases [5, 51–64]; MMPs have been implicated in physiological and pathological angiogenesis because of their fundamental nature in ECM metabolism and remodeling. During the onset of angiogenesis, this basement membrane matrix is degraded by proteinases to allow endothelial cell to migrate and various angiogenesis promoters and inhibitors such as growth factors, chemokines, growth factor receptors, adhesion molecules, and apoptosis mediators to be released from ECM [65–67].

#### 3.2.1. MMPs as Regulatory Molecules of Vascular Wall Resident Stem Cells

VW-PCs are capable of differentiating into pericytes and smooth muscle cells (SMCs) [68, 69]. Pericytes synthesize basement membrane matrix proteins, proteoglycans, such as decorin, biglycan, versican, aggrecan, and fibronectin and various collagens [70]. Tightly wrapped around the vessels, pericytic MSCs interact with another critical regulator of the vascular environment, the vascular basement membrane (VBM) [71, 72]. The VBM is a specialized extracellular matrix that surrounds the blood vessels of the body and is regulated through a control system involving proteases, which alter and degrade the matrix, and protease inhibitors, which maintain and protect the VBM from disruption. This interplay between proteases and protease inhibitors as well as its effects on the VBM profoundly influences vessel stability and, hence, many physiological and pathological processes, such as aneurysmal disease [73–77]. The pericyte–EC interface is rich in fibronectin deposition and contains tight and gap junctions as well as N-cadherin and b-catenin-based adherens junctions [78]. Fibronectin is concentrated at the pericyte–EC interstitium and its degradation by proteolytic enzymes such as MMPs gives rise to biologically active fragments [78]. Among these, a 45 kDa fibronectin fragment inhibits EPCs proliferation and stimulates pericyte and SMC proliferation, suggesting a role for this fragment in vessel maturation [79].

MMPs are probably the most important family in ECM remodeling and it is known that the cleavage of ECM liberates angiogenic factors [80–83]. SMCs can constitutively express and secrete MMP-2, and expression and secretion of MMP-9 are inducible in SMCs under the control of NF-kB; they express MMP-7 and MMP-3. Moreover, MMPs released by leucocytes and convected circulating plasma MMPs represent other important sources of MMPs in the arterial wall. SMCs are, in parallel, the main source of tissue protease inhibitors and also the possible target of blood-borne protease zymogens convected through the wall, retained or not, and directly or indirectly activated on contact with the SMCs [84, 85]. They also constitutively express and secrete several serine proteases, such as tissue-type plasminogen activator (t-PA), for which expression can be enhanced by numerous stimuli [86, 87]. Thus, in the vascular wall, SMCs are the main source of TIMPs and of several serpins, such as plasminogen activator inhibitor-1 and protease nexin-1 (PN-1) and probably cysteine inhibitors (cystatin) [88]. MMP-9 can convert normal nonangiogenic islets into angiogenic islets. More recently, it was reported that ectopic expression of Homeobox C11 (HOXC11), which is normally restricted to the SMCs of lower limbs vessels, in carotid arteries, aortic arch, and descending aorta, results in drastic vessel wall remodeling including elastic laminae fragmentation, SMC loss, and intimal lesion formation [89, 90]. These results suggest direct transcriptional control of two members of the matrix MMPs family, including MMP-2 and MMP-9 that are known as key players in the inception and progression of vascular remodeling events.

Many evidences have shown that the influence of a particular MMP may depend on the vascular bed analyzed or on a particular type of EPCs and its related receptor, and biophysical parameters (substrate elasticity, cell stiffness or cell shape, and vascular ischaemic injuries) can also promote the release of the serine proteases cathepsin G (catG) and neutrophil elastase (NE) and the secretion of the collagenase. MMP-8 and MMP-9 initiate a cascade of events including inactivation of retention factors, release and activation of mobilizing factors and cytokines, ECM degradation and remodeling with breakdown of cell-matrix interactions, and also breakdown of cell-cell contacts, ultimately resulting in stem cell egress; moreover, the reduction of endogenous protease inhibitors may also contribute to the highly proteolytic activity [91–93].

MMPs are also related to mitogenesis and migration of SMCs [92]. In in vivo studies, MMP-3 knockout mice reduced neointima formation after carotid ligation and also attenuated SMC migration into wound [94]. SMCs are important both to promote arterial remodeling and to modify vessel diameter and/or wall thickness to ensure adequate tissue perfusion [95].

In presence of VEGF, arterial wall resident cells became round-shaped, resembling ECs, and part of the cells acquired CD-31, VE-cadherin, and von Willebrand factor expression, whereas when they are cultured with TGF*β*-1 or platelet-derived growth factor-BB (PDGF-BB) adopted a rather elongated phenotype, similar to that of SMCs, and part of the cells acquired anti-*α*-smooth muscle actin (ASMA) and calponin [96]. VEGF also induces the expression of Notch1 through PI3K/AKT pathway in cultured ECs [97]. The roles of Notch include the differentiation in both EPCs and SMC via activation of transcriptional CBF-1/RBP-J*κ*-dependent and independent pathways and transduction of downstream Notch target gene expression [98, 99]. These angiogenic factors can induce differentiation from progenitor in media to EPCs and SMCs [9].

Recently it has been shown that pericytes are able to detach from the vascular wall and contribute to fibrosis by becoming scar-forming myofibroblasts in many organs including the kidney. At the same time, the loss of pericytes within the perivascular compartment results in vulnerable capillaries which are prone to instability, pathological angiogenesis, and, ultimately, rarefaction such as aneurysmal disease [100, 101].

Based on these evidences, we could affirm that MMPs may play a central role to regulate the activity of the VW-PCs by increasing the biodisponibility of main proangiogenic factors. Another role of MMPs is to promote the differentiation and migration of fibroblast and resident vasculogenic progenitors critically involved in vascular repair by remodeling of ECM [102]. MMPs contribute to VW-PCs during the progression of arterial aneurysms and participate in all crucial stages of this degenerative disease.

#### 3.2.2. Vascular Wall Resident Stem Cells in Natural History of Arterial Aneurysms: A Debate Still Open

As widely known, the pathogenesis of aneurysm involves inflammation, protease activation, ECM remodeling, and SMC dysfunction and apoptosis leading to the weakness of the vessel wall and arterial expansion under the influence of blood pressure [34]. Aneurysm complications, as rupture, dissection, and distal embolization, are frequent and with a high morbidity rate and an increase with the diameter of the vessel [103, 104].

Clinically, guidelines recommend surgical treatment for large aneurysms and monitoring for smaller aneurysms [103]. However, a significant number of small aneurysms, falling outside the criteria for surgical treatment, undergo complication development [104]. The identification of small aneurysms at increased risk of complications may improve the morbility and morbidity associated with this disease.

### 3.3. MMPs and Arterial Aneursysms

An association between arterial aneurysms and MMPs has been described in both central [58, 105, 106] and peripheral arterial diseases [107–118]. MMPs regulate extracellular structural proteins and tissue remodeling and are involved in several vascular diseases [4, 51, 119]. We have documented a significant correlation between age, median size of aneurysms, and plasma levels of both MMP-9 and neutrophil gelatinase-associated lipocalin (NGAL) in both central and peripheral aneurysms [32]. Degradation of ECM by MMPs allows the migration of vascular smooth muscular cells from the medial vascular layer to the intimal layer [51, 120–124]. These proteinases, degrading elastin, can induce a compensatory fibrosis and inflammation with destruction of all major matrix components, excessive distension, and rupture [125, 126]. Several cytokines and growth factors including IL-1a and b, IL-2, IL-17, insulin like growth factor-1, transforming growth factor alpha (TGF-*α*), and tumor necrosis factor alpha-a (TNF-*α*) can induce MMPs and NGAL, a marker of neutrophil activation that can modulate MMP-9 activity [58].

#### 3.3.1. Vascular Wall Resident Stem Cells and Aneurysms: Positive and Negative Effects

The role of VW-PCs in aneurismal formation is relatively unknown and remains controversial. Witte et al. showed that VW-PCs present intracytoplasmatic vacuoles as a sign of their inherent capacity to form a capillary lumen. It depends on local environment whether these cells undergo a differentiation or necrosis; maybe these cells undergo necrosis when red blood cells penetrate into their intracytoplasmatic vacuoles [127]. VW-PCs express STRO-1, c-Kit, and CD34 and, in response to tissue injury, can differentiate into SMCs and fibroblasts suggesting an active role in a repair and remodeling process [128]. C-kit cells can induce the secretion of angiogenic cytokines such as VEGF stimulating their proliferation and differentiation into ECs and MSCs [128].

The basic phenomena in the pathogenesis of arterial aneurysms are degradation of ECM components with increased MMPs and loss of structural integrity of the arterial wall [129, 130]. These pathologic changes are associated with chronic inflammation of aortic walls, where resident vascular SMCs and infiltrating macrophages release MMPs, particularly MMP-2 and MMP-9 [131, 132]. MSCs have also been reported to upregulate elastin and downregulate collagen gene expressions in fibroblasts and are known to participate in remodeling associated with vascular injury in a variety of settings [133, 134]. In arterial aneurysms, the medial fiber network is impaired, SMC number diminishes, and inflammatory cells invade the expanding vascular wall. The ECM alteration in the aortic wall depends on the balance between ECM synthesis from vascular SMCs and protease production by SMCs and inflammatory cells. As previously described, VW-PCs can be mobilized from adventitia to the media and differentiate to SMC in cases of injury or damage of the arterial wall cells in order to replace them. Moreover, the chronic exposition to inflammatory conditions such as natural history of aneurysmal disease [135–137] can determine failure of SMC recruitment and migration along developing vessels can lead to vascular instability and regression, an event that is likely due in part to the ability of these cells to secrete and organize extracellular matrix-containing basement membranes and elastin [18, 138–140]. In this view, human autopsies have demonstrated the presence of CD34+Sca1+CD133− cells within neointimal lesions and the adventitia of atherosclerotic plaques, which may be a source of endothelial and vascular smooth muscle cells that form atherosclerotic lesions [141–144]. Recently, Tigges et al. and other groups reported that adventitial multipotent pericytes participate in the restenotic response in mice with femoral arterial injuries [40, 145]: pericytes are increased in adventitia in response to vascular injury and contribute to restenosis in injured arteries. Pericytes have mesenchymal stem cell like features and are potentially an important cellular source that contributes to intimal hyperplasia in rat aortic allograft models with transplantation-derived arteriosclerosis [146, 147]. Many factors including cytokines such as TNF-*α*, IL-1, IFN-*γ*, and toxins of infectious agents and hypoxia can stimulate the release of many growth factors by MSCs, including EGF, FGF, PDGF, TGF-b, VEGF, hepatocyte growth factor (HGF), insulin growth factor-1 (IGF-1), angiopoietin-1 (Ang-1), keratinocyte growth factor (KGF), and stromal cell derived factor-1 (SDF-1) [148, 149]. These growth factors, in turn, promote the development of fibroblasts, endothelial cells, and tissue progenitor cells, which carry out tissue regeneration and repair.

Thus, VSMCs, the predominant cell type of the media, are capable of robust proinflammatory responses to diverse stressors. The multiple cytokines and chemokines produced within the media can profoundly affect macrophage and T cell function; on the other hand, VSMCs and the ECM are able to have significant anti-inflammatory properties. The balance between the pro- and anti-inflammatory effects of VSMCs and their extracellular matrix versus the strength of the inciting immunologic events determines the pattern of medial pathology. Limitations on the extent of medial infiltration and injury defined as “medial immune privilege” are typically seen in arteriosclerotic diseases, such as atherosclerosis which is the first step of aneurysmal disease. Conversely, the breakdown of medial immune privilege that manifests as more intense leukocytic infiltrates, loss of VSMCs, and destruction of the extracellular matrix architecture is a general feature of certain aneurysmal diseases and vasculitides [150, 151]. Tissue injury is always associated with the activation of immune/inflammatory cells, not only macrophages and neutrophils but also adaptive immune cells, including CD4+ T cells, CD8+ T cells, and B cells, which are recruited by factors from apoptotic cells, necrotic cells, damaged microvasculature, and stroma [152, 153]. Insufficient inflammatory cytokines during chronic inflammatory sites, however, could stimulate MSCs to produce chemokines and tropic factors in absence of sufficient immune inhibitory factors. As such, chronic inflammation may lead MSCs to protract the disease recovery or even worsen the disease course such us in aneurysmal disease [154, 155].

Specifically, progenitor cells can contribute to calcification as bone marrow (BM) contains both osteoblast and osteoclast precursors termed as osteoprogenitors (OPs) associated with bone remodeling [156]. This novel mechanism was named “circulating cell theory.” The bone marrow derived cell population may seed the arteries and contribute to disease or repair [156, 157]. Another common mechanism that can explain the recruitment of circulating OPs in arteries is homing; in response to stress signal, injury, inflammation, repair, or abnormal cytokine signaling, circulating cells cross the endothelium and invade the target tissue [157, 158]. The endothelial phenotype selectively modulates bone marrow derived stem cells homing: indeed different endothelial phenotypes hold functional differences. As an example, coronary artery endothelium enables the fastest bone marrow stromal cells integration. Transmigration requires the interaction of vascular cell adhesion molecule-1, very late antigen-4, *β*1 integrins, MMPs secretion, and cytokines [159, 160]. Moreover pericytic myofibroblasts expressed BMP-2, a powerful bone morphogen. Recently it was hypothesized that MSC might play a role in the pathogenesis of atherosclerosis, and it was demonstrated that, under particular conditions, MSC in culture acquires an osteoblastic phenotype via the activation of the Wnt pathway [161, 162]. In hyperlipidemic rats treated with angioplasty to have a vascular damage, MSC started the vessel wall remodeling and triggered calcification, mediated by paracrine BMP-2 [163, 164], which is considered one of the main mediators in the differentiation of MSC (and others) along the osteoblastic lineage. The putative role of pericytes as a “reservoir” of progenitor cells, as well as their potential to differentiate into several cell types, including osteoblasts, is well known [165, 166] and many evidences have been adduced that pericytes can undergo chondro and osteogenic differentiation [167–169]. This represents an interesting example of indirect stimulus towards calcification mediated by the synergic cross-talk between different cells of the vessel wall.

Moreover, as described previously, VW-PCs may reduce aneurysmal degeneration through the suppression of MMP expression [36]. Furthermore, VW-PC may facilitate tissue damage by differentiating into inflammatory cells. VW-PC may represent a reservoir for the localized replenishment of aneurysm wall macrophages [33, 36]. Thus, depending on the local environment and paracrine manner via cytokines and growth factors, the VW-PC could contribute to ongoing inflammation and aneurysmal degeneration or accelerate vascular repair [170].

#### 3.3.2. MSCs Application in Cardiovascular Regenerative Therapy: The State of the Art

VW-PCs, circulating EPCs, and umbilical cord blood cells present multiple important clinical interests. EPCs could be used to treat diverse vascular disorders because of their high migratory potential through blood and their capacity to differentiate into new endothelial cells that can contribute to promoting neoangiogenesis and endothelium repair at distant damaged tissues/organs [171, 172]. In vivo induction of mobilization of bone marrow-derived EPCs into peripheral circulation or activation of EPCs resident in vascular wall of damaged peripheral tissues could represent promising strategies to promote vascular repair of injured areas. It has been observed that EPCs were able to give rise to the endothelial cells that incorporated into the endothelial layer and this led to a reduction of the lesion size [173].

Studies have shown that the effects of MSCs upon damaged regions have been proven, causing the inhibition of local immune response, preventing excessive fibrosis, apoptosis, and inducing mitosis in intrinsic cellular progenitors [174]. These immunomodulating effects are caused by reducing the functions of B and T lymphocytes and natural killer cells, affecting the function of dendritic cells [175, 176]. Moreover, MSCs cause a low immunogenic effect, even upon models or patients with different human leukocyte antigen (HLA), due to low expression levels of HLA-I and null expression levels of HLA-II [177–179]. Porcine models of myocardial infarction have further demonstrated the reparative potential of MSCs when administered* acutely* after injury [180–184]. The local injection of MSCs in a porcine model of myocardial infarction demonstrated not only the successful engraftment of locally injected MSCs but also their multiphenotypic differentiation. These are able to evolve into cells that have biologic characteristics of cardiac myocytes and endothelial cells. These findings were described along with improvement of cardiac function compared with untreated controls [185–187]. The ability of postnatal skeletal muscle to repair and regenerate itself on daily physical activity or injury is well documented. However, severe pathological conditions, such as compartment syndrome and muscular dystrophy, impede structural and functional recovery mediated by myogenic progenitors and require exogenous interventions to ameliorate the progression [188–191]. Transplanted pericytes, purified from human skeletal muscle, fat, pancreas, and placenta, regenerate human myofibers in cardiotoxin-treated and dystrophic mouse muscles more efficiently than do myoblasts or endothelial cells. In addition to structural regeneration, functional recovery was demonstrated in dystrophic mice treated with pericytes isolated from muscle biopsy specimens from not only healthy adults but also, surprisingly, patients with Duchenne muscular dystrophy [192–194]. There is a linear relationship between the outcome of treatment and the type of cells applied. Osteogenic, odontoblastic, and adipogenic progenitors have also recently been shown to originate from perivascular niches in vivo, in agreement with the robust osteogenic and adipogenic properties found in purified pericytes [195, 196]. These discoveries imply that pericytes can potentially be applied to bone regeneration, dental repair, and adipose reconstruction [197]. Higher therapeutic efficacy, including complete restoration of kidney function, was observed after infusion of cord blood (CB) MSCs/pericytes compared with regular bone marrow-derived MSCs. However, few donor cells were found in the restored area; also, it was shown in culture and in vivo that the observed renoprotective effects are mediated mainly by angiogenic and antiapoptotic factors secreted by the CB MSCs/pericytes [198, 199] and another source of stem cells is the umbilical cord itself [200–202]. In the perspective of cell therapies, the pericytes are mobilized and migrated toward the damaged cells, secreting high levels of antiapoptotic and angiogenic factors, such as vascular endothelial growth factor and keratinocyte growth factor. These findings suggest that pericytes can efficiently move to damaged sites and secrete growth factors that can play beneficial autocrine or paracrine roles in tissue and vascular repair [203–207]. MSCs are localized in the vascular niche in bone marrow but are also found as MSC-like cells around adult vessels (also termed pericytes and adventitial cells), and there is substantial evidence that they play a pivotal role in regulating blood vessel formation and function through multiple mechanisms such as vasculogenesis, arteriogenesis, and angiogenesis. Although MSCs or MSC-like cells have been safely used and do not pose the ethical concern of embryonic stem cells, their effects in clinical studies cannot be delineated to specific mechanisms. These might include different simultaneously acting MSC-induced mechanisms. Immunomodulation towards a more repair-friendly microenvironment, actual differentiation into vascular tissue, and paracrine or systemic release of vasculogenic, angiogenic, and/or arteriogenic-stimulating factors should in this respect be acknowledged. Additionally, the results of preclinical studies have been shown to depend not only on the model chosen and the endogenous repair capacity of the cardiovascular tissue in vivo, but also on cell source, administration route, timing of cell delivery, and cell dosage and with these specific homing and retention mechanisms. Clinical studies on necessarily heterogeneous patients add many variables (e.g., inflammatory and disease status, comorbidities, and concomitant medication) and may explain the differences in the results observed so far. MSCs markedly suppressed MMP gene expression in macrophages in vitro, MMP-2 activity ex vivo, and MMP activity in vivo and influenced TIMP-1 in vivo. Negative correlations between elastin content and MMPs were confirmed [32]. MSCs also decreased expression of inflammatory cytokines, including IL-6, MCP-1, and TNF-alpha which potentially may in turn lead to MMP upregulation in the aortic wall. This finding implies that MSCs might suppress the excess immunopathologic reactions in the aneurysmal vascular wall in a paracrine manner without direct cellular contact. MSCs from bone marrow have been reported to suppress dendritic cells, T cells, and natural killer cell activities in vitro, which may be attractive in this setting. Previous work has demonstrated that MSC mobilization and homing are induced by MMP-2, MMP-9, chemokines, or elastases. MSCs are also known to possess tropism for inflammation. Because aortic ECM degradation by MMP-2 and MMP-9 and chronic inflammation of the aortic wall induced by chemokines are essential features of AAs, MSCs that likely migrate toward MMPs and chemokines have an advantage for aortic aneurysmal cell therapy [208, 209].

## 4. Discussion

Pathogenesis of aneurysm commonly involves inflammation, MMPs activation, ECM remodeling, and VSMC dysfunction and apoptosis, which ultimately lead to the weakening of the vessel wall and arterial expansion under the influence of mechanical forces. Rupture, dissection, and distal embolization are frequent and highly morbid complications of aneurysm [210]. The degenerative remodeling seen in arterial aneurysms can result from a combination of excessive destruction and insufficient repair; when tissue is injured, inflammatory cells infiltrate the injured area to clear damaged or dead cells and degraded proteins. Evidences have shown that SCs play an important role in tissue repair and regeneration: SCs can recruit and stimulate the proliferation of resident SCs, creating a favorable microenvironment for vascular repair [211]. Studies recently have shown VW-PCs in the adventitia of ApoE-deficient mice and these progenitors contributed to experimental atherosclerosis and did not originate from the bone marrow [212, 213]. VW-PCs have been also isolated from the thoracic and abdominal aortas of humans: it was found that a subpopulation of EPCs was organized in a completely hierarchical manner in a distinct zone of vascular wall which was named as “vasculogenic zone” [16]. As mentioned above, CD34(+) cells have paracrine activity, can secrete vascular endothelial growth factor, and can promote neovascularization.

In cases of chronic inflammation such as arterial aneurysms, the local proangiogenic environment caused by activation of MMPs would induce the mobilization of local VW-PCs and tissue-resident EPCs faster than that of the circulated-EPCs or BM-EPCs and the presence of multipotent SCs at sites of aneurysm and dissection formation that can further differentiate into SMCs suggests the existence of an active repair process involving SCs. VW-PCs are relevant for the regeneration of vasa vasorum, a part of vessels which provide the blood supply for the outer layers of the vascular wall, such as the adventitia and neighbored parts of the tunica media including the “vasculogenic zone,” where the VW-PCs reside.

VW-PCs not only may promote vascular repair by differentiating into vascular SMCs and fibroblasts, but also may facilitate tissue damage by differentiating into inflammatory cells. Active MMPs can induce the secretion of angiogenic cytokines such as vascular endothelial growth VEGF and stimulate host SCs proliferation and differentiation. Each of these cell types has a different function and could lead to effective repair, maladaptive remodeling, or further arterial damage.

Other several mechanisms involved in arterial aneurysms pathophysiology are hemodynamic forces (share stress); these factors are important mediators of vascular remodeling promoting arterial ECs proliferation and migration and medial SMC proliferation resulting in adaptive enlargement and luminal tortuosity. Thus, VW-PCs are innately resistant to proaneurysmal environmental stresses such as reactive oxygen species production; VSMC-PCs significantly decreased expression of MMPs and were able to attenuate formation of elastase-induced arterial aneurysms [214]. MMPs are a family of zinc dependent proteolytic enzymes that degrade various components of ECM and mediate ECM remodeling in both physiological and pathological processes. Several works reveal that proteolytic activity of MMPs controls availability of active molecules such as growth factors [215]: MMPs play a critical role in vascular formation and remodeling through degrading vascular basement membrane and ECM proteins and modifying angiogenic growth factors and cytokines. Both vascular formation and remodeling are complicated processes including recruitment, migration, proliferation, and apoptosis of vascular cells consisting of stem/progenitor cells, ECs, VSMCs, and other mural cells. ECM degradation and remodeling indispensable to vascular structure alterations highlight MMP functions in VSMC behaviors. MMP-2, MMP-9, MT1-MMP, MMP-3, MMP-1, and MMP-7 have been recognized in vascular tissue and play pathogenic roles in vascular remodeling via regulating VSMC behaviors [216].

Early outgrowth EPCs have limited capacity for population doubling and induce only transient angiogenesis; late outgrowth EPCs can expand to more than 100 population doublings. Early outgrowth EPCs exert an angiogenic effect mainly by secretory products, whereas late outgrowth cells were thought to produce the effect by direct engraftment. Among those were MMP-9, IL-8, macrophage migration inhibitory factor, various cathepsins and protease inhibitors, S100 proteins A11, A8, and A4, plasminogen activator inhibitor-2, and apolipoprotein E as well as a potent proangiogenic and prosurvival factor, and thymidine phosphorylase [217, 218].

It is possible to assume that the VW-PCs act, with different functions, in different phases of the natural history of aneurysms. In the early stages, under the auspices of the various growth factors released by the action of MMPs, the VW-PCs were associated with compensatory mechanisms that vessels oppose to lesional phenomena of their wall; in the later stages, VW-PCs may actively participate and contribute to the formation of the aneurysm, through the gradual and definitive calcification and loss of function of the arterial wall, and its rupture and dissection.

Stem cells are quiescent and reside in “stem cell niches” of the vessel wall but they become activated by insult stimuli, for example, endothelial injury by angioplasty or aneurismal development. If damage is moderate, the laminar flow will stimulate stem cells to differentiate into ECs to maintain the vessel integrity. When severe damage or atherosclerotic lesion occurs, locally the disturbed flow is induced, resulting in stem cell differentiation towards SMCs, which accumulates within the intima [219, 220].

The existence of VW-PCs provides an exciting prospect to directly manipulate local responses within the vasculature, as it has already happened, in a similar way, in cell therapy for critical limb ischemia [220]. In fact, several approaches such as site specific delivery and generating MMP inhibitors with increased selectivity are thought to be helpful for MMPs-targeted therapy.

It could be concluded that, therapeutically, the benefit to address VW-PCs at sites of arterial aneurysms may be the possibility to predict the natural history of arterial aneurysm and frame the developmental stage of disease, studying also the behavior of the cells involved in the inflammatory process characterizing the aneurysm.

Then, addressing the specific MMPs involved in VW-PCs activities, by means of specific antiproteases drugs, may prevent that the initial compensatory mechanism will be replaced by the anomalous degenerative mechanism which leads to aneurysm formation.

## Abbreviations

AAA: Abdominal arterial aneurysms
ASMA: Antialpha smooth muscle action
BM-EPCs: Bone marrow-derived endothelial progenitor cells
CAD: C-terminal activation domain
ECM: Extracellular matrix
ECs: Endothelial cells
EGF: Epithelial growth factor
EPCs: Endothelial progenitor cells
FIH-1: Factor inhibiting HIF-1
FSP1: Fibroblast marker
G-CSF: Granulocyte-colony stimulating factor
GM-CSF: Granulocyte macrophage-colony stimulating factor
HGF: Hepatocyte growth factor
HLA: Human leukocyte antigen
HIF-1: Hypoxia-inducible factor-1
HOXC11: Homeobox C11
IFN-*γ*: Interferon gamma
IL-1: Interleukin 1
KGF: Keratinocyte growth factor
MAPK: Mitogen activated protein kinase
MCP-1: Monocyte chemoattractant protein-1
MMPs: Metalloproteinases
MSCs: Mesenchymal stem cells
MT1-MMP: Membrane type-1 metalloproteinases
NGAL: Neutrophil gelatinase-associated lipocalin
PAR-1: Protease-activated receptor
PDGF: Platelet-derived growth factor
PDGF-BB: Platelet-derived growth factor-BB
SCs: Stem cells
SDF1-*α*: Stromal cell derived factor 1-alpha
SM22: Smooth muscle cell marker 22
SMCs: Smooth muscle cells
TGF-*α*: Transforming growth factor alpha
TGF-*β*: Transforming growth factor beta
TIE-2: Tyrosine kinase with immunoglobulin-like and EGF-like domains 2
TNF-*α*: Tumor necrosis factor alpha
VEGF: Vascular endothelial growth factor
VBM: Vascular basement membrane
VEGFR2: Vascular endothelial growth factor receptor 2
VSMCs: Vascular smooth muscle cells
VW-PCs: Vascular wall resident progenitor cells.
